# A multilayered urban tree dataset of point clouds, quantitative structure and graph models

**DOI:** 10.1038/s41597-023-02873-x

**Published:** 2024-01-04

**Authors:** Hadi Yazdi, Qiguan Shu, Thomas Rötzer, Frank Petzold, Ferdinand Ludwig

**Affiliations:** 1 School of Engineering and Design, Technical University of Munich, Munich, 80333 Germany; 2 School of Life Sciences, Technical University of Munich, Freising, 85354 Germany

**Keywords:** Forestry, Scientific data

## Abstract

The significance of urban trees in promoting human health and well-being has been amplified by urbanization and the climate change effects. Simultaneously, advancements in remote sensing techniques have enhanced the opportunities for studying urban trees. The TreeML-Data has been compiled to support these efforts. It consists of labelled point clouds of 40 scanning projects of streets in Munich, 3,755 leaf-off (scans in winter) point clouds of individual trees, quantitative structure models (QSM), tree structure measurements, and tree graph structure models of these trees. The dataset offers valuable data for generating and evaluating models in various scientific disciplines, which include remote sensing, computer vision, machine learning, urban forestry, urban ecosystem, green architecture, and graph analysis. To ensure its quality, the tree structure measurements and QSM have been crosschecked. For instance, the tree diameter at breast height (DBH) in the sample dataset exhibits a deviation of approximately 1.5 cm (4.3%) when compared to manual measurements. In conclusion, the quality checks confirm its reliability for subsequent studies when compared to manual measurements.

## Background & Summary

In light of the growing trend of urbanization and the impact of climate change, the concept of urban green infrastructure (UGI) has gained prominence as a mean to enhance human health and well-being in urban areas^1^. Given their significant role in UGI, it is crucial to thoroughly examine and research trees. Hence, maintaining an updated and precise inventory of individual urban trees is essential for conducting comprehensive urban forestry studies and supporting decision-makers in strategic planning processes^2^. Furthermore, on a smaller scale, the tree canopy design and maintenance in contrast to the surrounding buildings can have profound effects on human well-being, including impacts on urban microclimate, building energy consumption, and the provision of ecosystem services^3^. Professional arborists usually have been responsible for compiling tree inventories, capturing a diverse range of variables such as location, species, vitality status, height, and diameter at breast height^4^. These variables are crucial for supporting various decision-making and planning activities, including prioritizing maintenance, assessing biodiversity, species, and size class distribution, and estimating ecosystem services (ESS)^5,6^. Researchers also rely on repeated tree measurements and systematic monitoring to analyse trends in street tree population and growth, including changes in composition and mortality rates^7^. However, due to the time and quality efficiency of data collection by arborists and the intensive human work involved, alternative methods are being employed to conduct much more up-to-date tree documentation in a shorter time, such as remote sensing techniques^7,8^. These novel approaches help to ensure the speed, quality, and efficiency of inventory updates. Therefore, the utilization of high-resolution remote sensing data has emerged as a novel approach for accurately mapping individual trees within urban areas^9–11^. Over the past decade, the widespread adoption of Terrestrial LiDAR Scanning (TLS) technology has facilitated the detailed documentation of objects using point cloud data^12^. This progress has been extended to tree surveys, as remote sensing experts have applied these technologies to capture accurate tree structure. The topologies of the tree trunks and branches can be abstracted from a 3D point cloud geometry^13^. Shu *et al*.^14^ coined the term Tree Information Modelling (TIM) and summarized different approaches: For example, Raumonen introduced TreeQSM^15^ to reconstruct tree topology, while PypeTree^16^ reconstructes trunks and branches based on skeleton curves. SimpleTree^17^ utilizes voxel-grid-based techniques to create cylindrical tree models, and AdTree^18^ employs a skeleton-based approach, fitting cylinders to point cloud models of individual trees. Based on Quantitative Structure Models (QSM) of trees, we propose the use of a graph data structure as a novel approach in tree studies, opening up new research opportunities. The graph data structure is a data format composed of nodes and edges, where nodes represent samples containing various features, and edges represent connections between nodes. The application of the graph data structure spans across different fields of study^19^. In recent years, there has been significant progress in employing machine learning methods, such as deep learning and convolutional neural networks, to analyse graph data structures and enhance prediction accuracy for non-linear data^20,21^. To develop a machine learning prediction model for tree canopy growth based on local environmental factors (TreeML-model), we use the knowledge gained from previous remote sensing tree measurement studies. We have collected precise tree structure data and quantitative structure models to gather a high-quality dataset for our TreeML model. Furthermore, to facilitate further research and projects, with this paper we publish the TreeML dataset, that researchers can use in their own studies. And we make our methods, codes, and data publicly available to facilitate easy access for future investigations.

## Methods

**Fig. 1:**
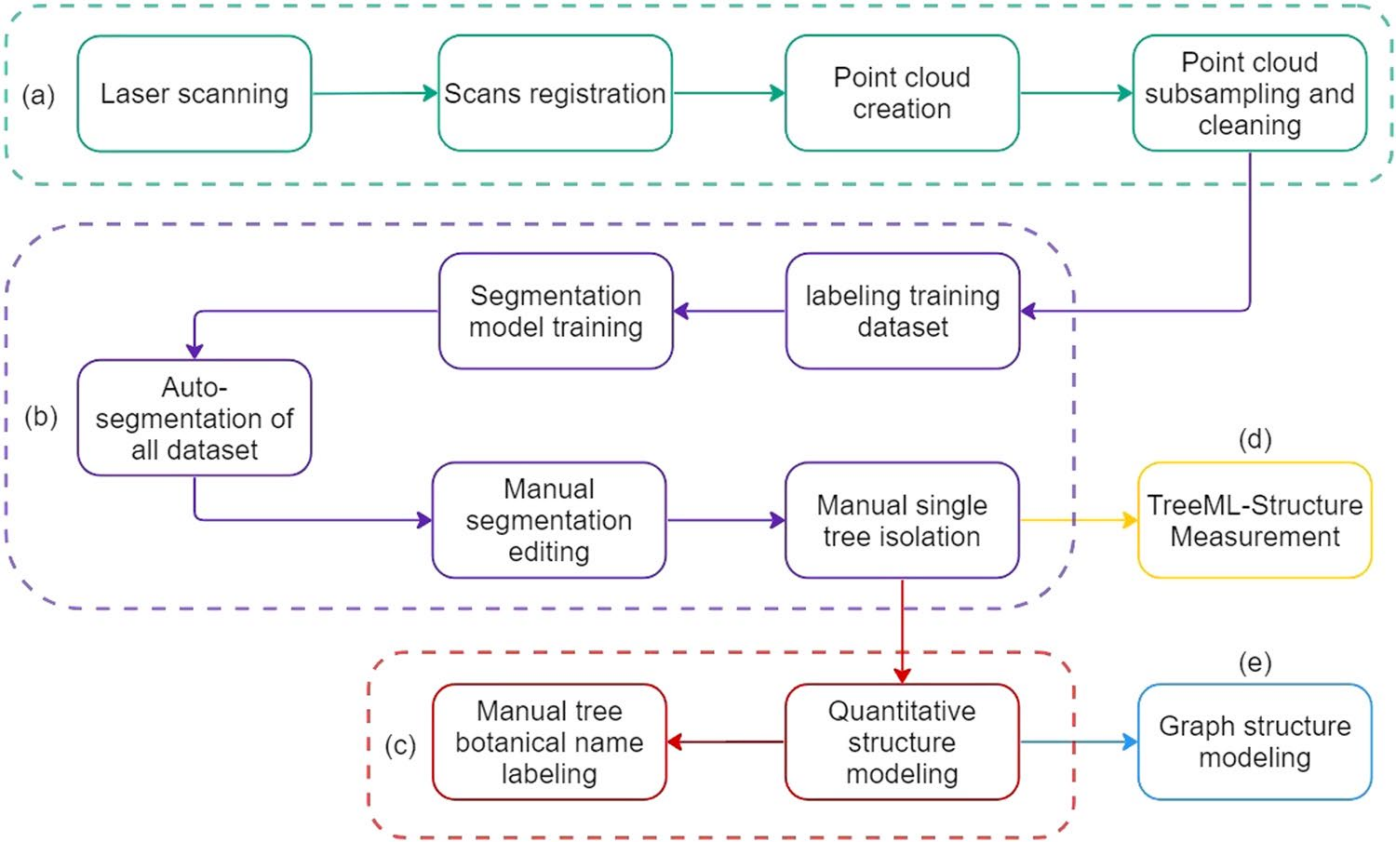
The method framework of the TreeML-Data. (**a**) Laser scanning and point cloud creation. (**b**) Semi-automated point cloud segmentation and tree isolation. (**c**) Quantitative structure modelling and tree structure measurement. (**d**) TreeML-Structure Measurement (TreeML-SM). (**e**) Graph structure modelling.

The^22^ preparation is divided into five main steps as shown in Fig. 1. In the first step (a), the streets are scanned to create point clouds. Subsequently, subsampling and cleaning techniques are applied to focus on the project area of interest. The second step (b) involves semi-automated point cloud segmentation and tree isolation. In this step, a segmentation model is trained using a labelled training dataset to accurately segment the entire project areas. Manual correction is then performed to refine the segmentation results, ensuring a high-quality dataset for further analysis. The third step (c) entails quantitative structure modelling (QSM). QSM reconstruction techniques, as proposed by Raumonen^15,23^, are utilized to model the branch-level structure of the trees. Additionally, the botanical names of the trees are manually labelled by observation in this step. In fourth step (d), we developed TreeML Structure Measurement (TreeML-SM) to measure the overall tree structure in the point clouds data. The final step (e) focuses on graph structure modelling, where a graph model dataset is generated for each tree based on its QSM data. This step allows for a comprehensive representation of the tree’s structural characteristics.

### Laser scanning and point cloud creation

**Fig. 2:**
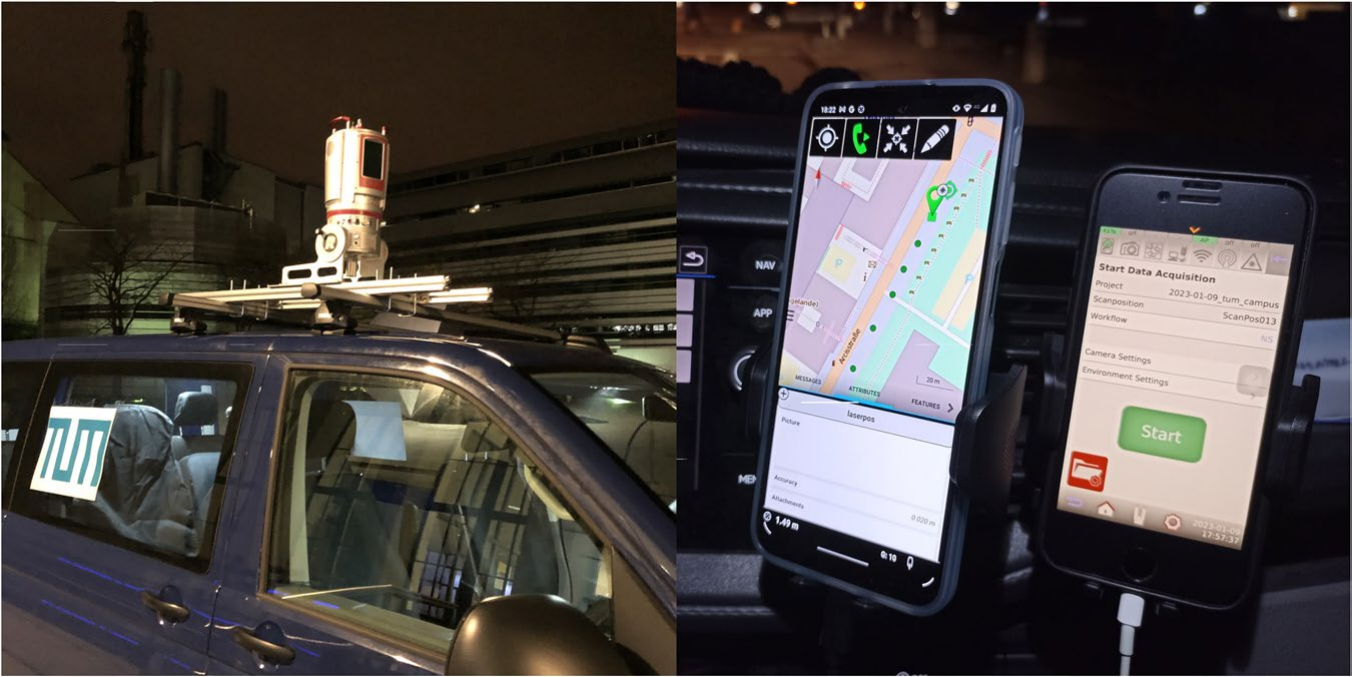
For mobile laser scanning setup a Riegle laser scanner was mounted on the roof of a car (left). Additionally, two cellphones were installed inside the car to control the laser scanner and GPS antenna (right).

For data gathering, the Riegle VZ-400i TLS laser scanner was used (Fig. 2 left). The scanning process involved a “stop-and-go” approach, where the vehicle would stop at each scan position before moving to the next. To accurately capture the global location of each scan, a Leica Zeno FLX100 precise GPS antenna was attached to the laser scanner. Both the laser scanner and GPS antenna were controlled remotely from inside the car (Fig. 2 right). The initial resolution setting on the laser scanner was set to “Panorama40” (40 [mdeg]), resulting in a varying number of points per scan, ranging from 10,000,000 to 25,000,000 points. The GNSS records demonstrated an accuracy of approximately 2 cm in most cases.

**Fig. 3:**
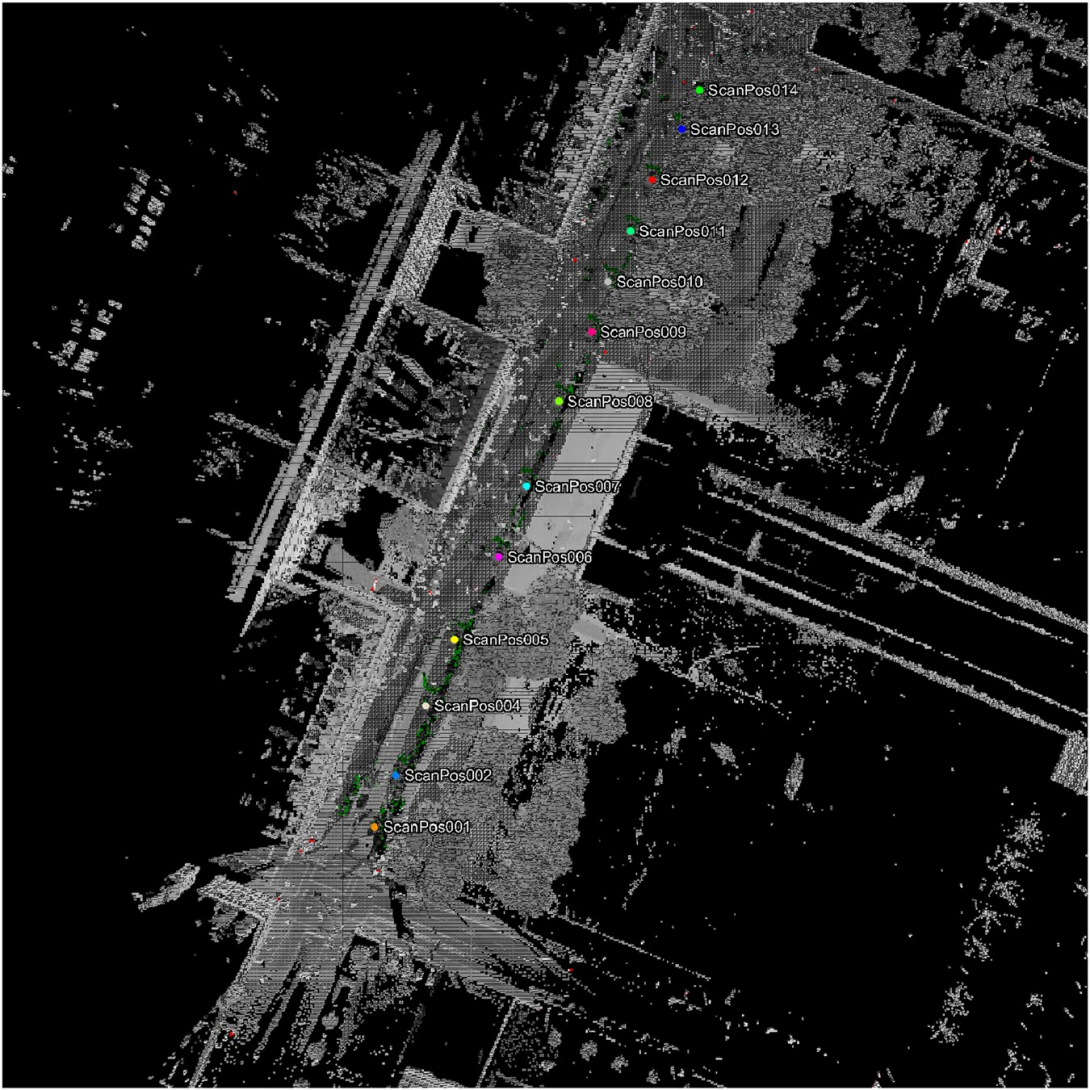
A sample scanning project (2023-01-09_tum_campus) in RiSCAN Pro software. The figure displays the outcomes of registration and location adjustment. It visualizes the scan positions and the initial point cloud before cleaning and subsampling.

Scan registrations and adjustments were performed using RiSCAN Pro 2.16.1, utilizing the GPS locations as a reference (Fig. 3). The scanning projects were registered within their project coordinate system, which can be converted to geocentric coordinate system and geographic coordinate system using the provided transformation matrix for each project (available in figshare repository^22^) and a Python script in GitHub repository (https://github.com/hadi-yazdi/TreeML-Data). The transformation matrix, represented as a 4 × 4 matrix, is applied to the input coordinates (*X*_*i*_, *Y*_*i*_, *Z*_*i*_) to calculate the geocentric coordinates (*X*_0_, *Y*_0_, *Z*_0_) following the methodology outlined by Wolberg^24^ (see Eq. 1).

1 $$\left[\begin{array}{c}{X}_{0}\\ {Y}_{0}\\ {Z}_{0}\\ 1\end{array}\right]=\left[\begin{array}{cccc}{a}_{xx} & {a}_{xy} & {a}_{xz} & {a}_{xt}\\ {a}_{yx} & {a}_{yy} & {a}_{yz} & {a}_{yt}\\ {a}_{zx} & {a}_{zy} & {a}_{zz} & {a}_{zt}\\ 0 & 0 & 0 & 1\end{array}\right]\cdot \left[\begin{array}{c}{X}_{i}\\ {Y}_{i}\\ {Z}_{i}\\ 1\end{array}\right]$$

With this matrix, the coordinates of each point in the point cloud can be transformed to a global coordinate system. The same methodology is employed to transfer the local project tree locations to global coordinate tree locations within the final dataset. Following the creation of each point cloud, the projects undergo subsampling using the Octree setting in RiSCAN Pro to achieve a point density of one point in each 2 cm. Additionally, the RiSCAN Pro is used to clean the projects by removing points outside of the focused scanning project area. This process ensures that the resulting point cloud is free from extraneous data.

### Semi-automated point cloud segmentation and tree isolation

Segmenting the point cloud data of trees and buildings can be a time-consuming task, especially when dealing with large datasets like the TreeML-Data. To address this challenge, a point cloud segmentation model was trained using a smaller portion of the dataset, enabling the segmentation of the entire dataset. The training dataset was labeled using CloudCompare 2.13 and consisted of three classes: “Tree”, “Building” and “Other”. The “Other” label was assigned to points that did not belong to trees or buildings. The PointNet++ segmentation model^25^ was employed for training, utilizing a GitHub repository titled “Tree segmentation using PointNet++” (https://github.com/murtiad/Tree_segmentation-using_PointNet). The training process was conducted on a cloud computer equipped with 8 CPUs, 32GB RAM, and an NVIDIA Tesla V100-PCIE-32GB GPU. After 30 epochs of training, the best-performing model achieved an evaluation accuracy of 97% and a best mean Intersection over Union (mIoU) of 94% (Table 1). These metrics demonstrate the effectiveness of the segmentation model in accurately classifying tree and building points within the dataset.

**Table 1:** The evaluation report of the best saved model of the segmentation model.

Best model evaluation					
Eval accuracy	Eval mean loss	Other IoU	Building IoU	Tree IoU	Best mIoU
0.970964	0.121596	0.931	0.953	0.939	0.940992

**Fig. 4:**
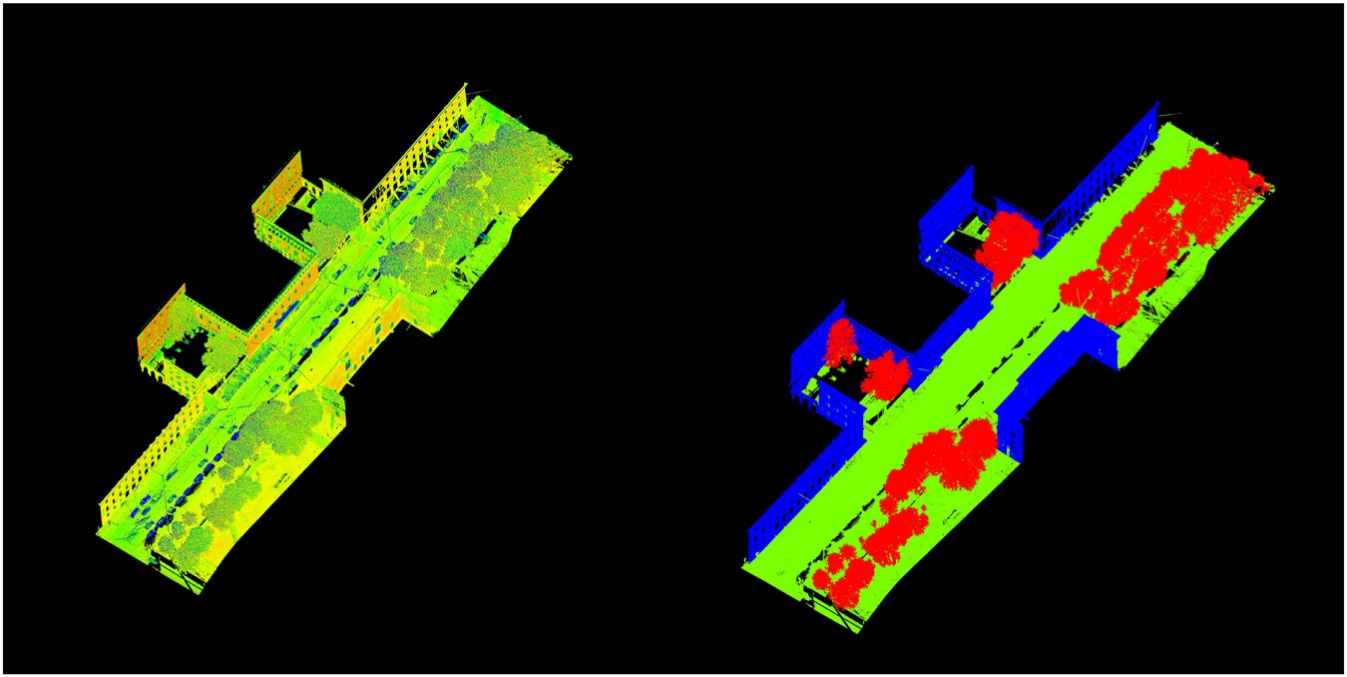
A point cloud visualization of a sample scanning project. Left side: Original point cloud before any labeling or annotations. Right side: The point cloud after being labeled using the PointNet++ trained model and undergoing manual corrections. The labels are Tree(red), Building(blue), and Other(green).

Following the training of the PointNet++ model, which achieved a high accuracy of 97%, we utilized the trained model to predict the labels for all projects in the dataset. Despite the high accuracy of the predictions, we conducted a thorough manual review of each project to ensure the dataset’s accuracy for subsequent steps. Figure 4 presents a sample of the results from this manual verification process with the three assigned labels: Tree (red), Building (blue), and Other (green). This figure exemplifies the high accuracy achieved after the manual editing, providing a reliable and precise dataset for further analyses and investigations.

**Fig. 5:**
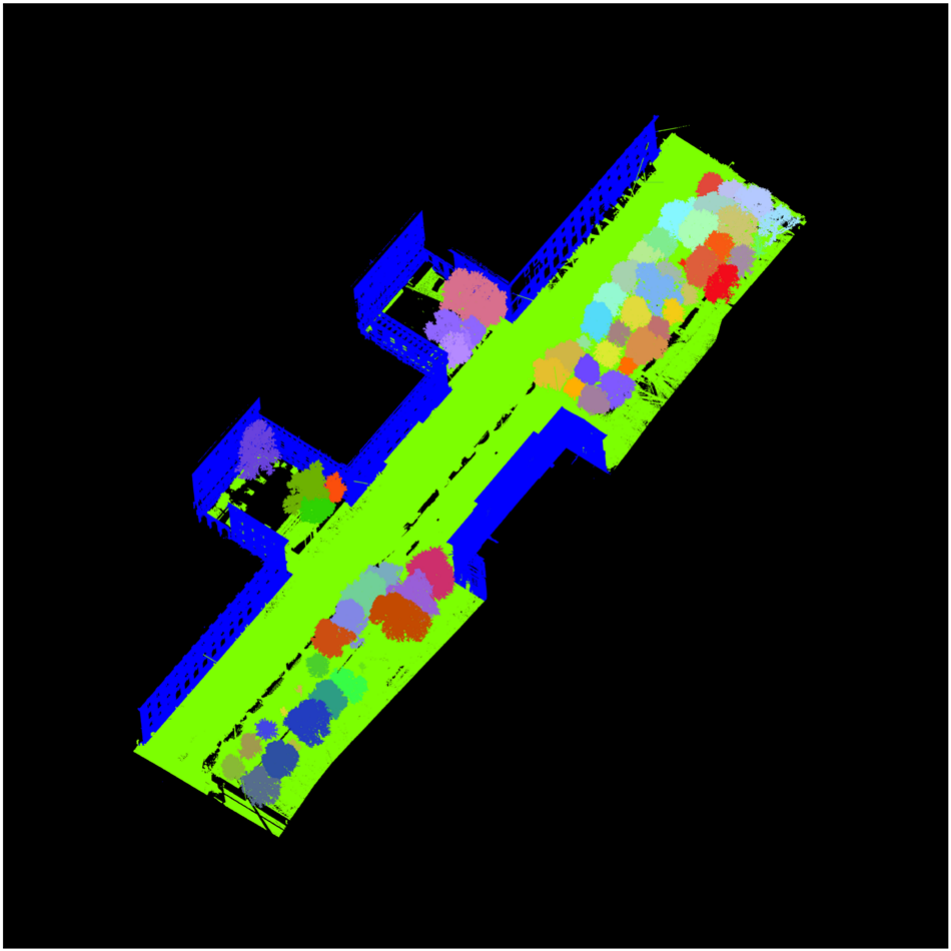
The dataset includes a labeled point cloud representation of 65 individual trees, along with buildings and other points.

In order to produce a high-accuracy dataset of tree canopy shapes, we manually isolated all trees in the dataset. This process proved to be time-consuming, requiring approximately 320 hours of work. To our knowledge no reliable automated methods for accurately isolating tree canopies and branches are available. Nonetheless the meticulous manual procedure ensured the generation of a valuable dataset for future studies. During this process, we encountered several trees with incomplete or insufficiently clean point clouds, which made them unsuitable for further processing. These trees were subsequently separated from the main dataset and saved as “trashtree” point cloud files to ensure the integrity and cleanliness of the primary dataset. Figure 5 depicts a labeled point cloud of a sample project with 65 trees. In addition to the trees, the dataset includes point clouds representing buildings and other objects, resulting in a total of 67 different objects.

### Quantitative structure modelling and Tree structure measurement

**Fig. 6:**
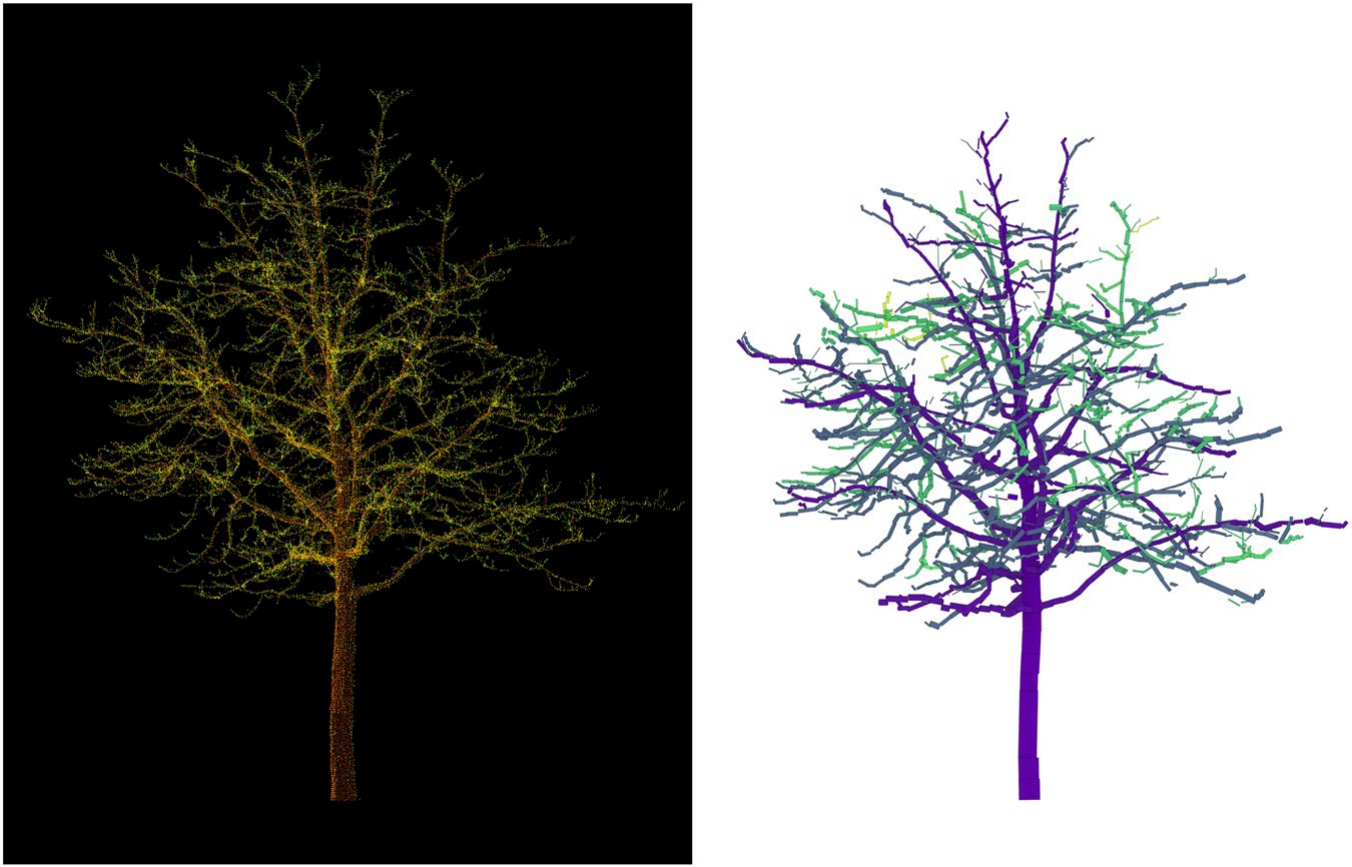
The figure illustrates the point cloud representation (left) and the corresponding Quantitative Structure Model (QSM) (right) of a sample tree from the dataset. The QSM depicts the tree’s branch structure, providing valuable information about the hierarchical organization of the tree’s branches.

In the subsequent step, we focused on generating the branch structure of each individual tree in the dataset using quantitative structure modelling (QSM) with the TreeQSM software^15^ implemented in MATLAB (https://www.mathworks.com/). To accomplish this, we referred to the TreeQSM GitHub tutorial and repository (https://github.com/InverseTampere/TreeQSM) for applying the method to our dataset. TreeQSM employs a cylinder fitting method to construct structure models of trees based on the available point cloud data. We conducted tests using 18 different configurations of patch sizes to reconstruct QSMs for each tree. Additionally, the QSM generation process for each configuration was repeated 15 times to mitigate the impact of random seed variations on QSM construction. To accelerate the QSM generation process for the dataset’s 3,744 trees, we employed five cloud computers with 45 GB of RAM and 10 virtual CPUs, effectively distributing the computations in parallel. This parallelization strategy significantly improved the processing efficiency and minimized the overall runtime. However, it is worth noting that TreeQSM encountered difficulties in modelling 11 trees within the dataset, resulting in crashes during the execution. Consequently, the final QSM dataset comprises 11 fewer trees compared to the original point cloud dataset. Figure 6 illustrates an example tree from the dataset, showcasing both the point cloud visualization (left) and an illustration of the corresponding QSM model (right). These visualizations provide a clear representation of the tree’s geometry and structure, allowing for detailed analysis and further studies.

**Fig. 7:**
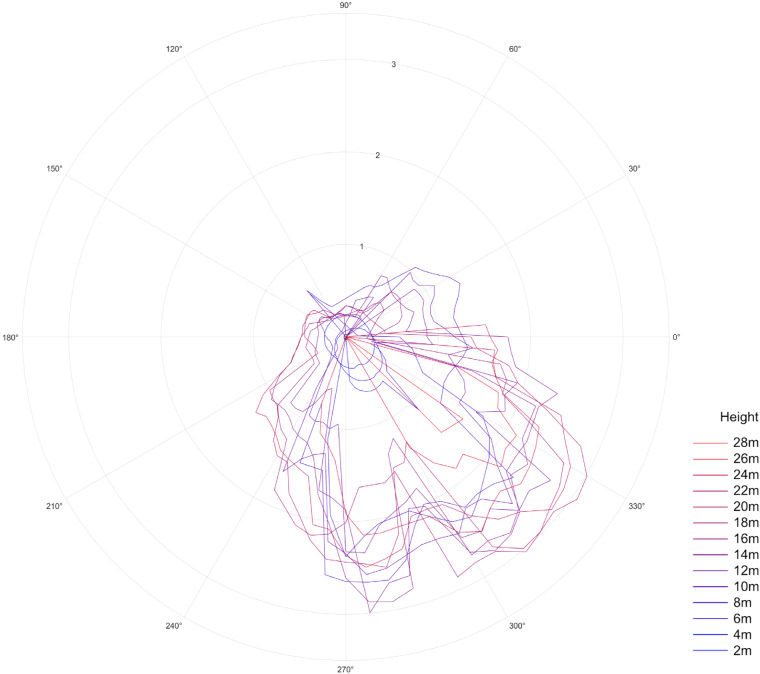
A diagram depicting the tree crown radius at each 5-degree interval and at 2-meter height increments.

In conjunction with the quantitative structure modelling (QSM) process, we also extracted tree structure measurements from TreeQSM and organized them in a tabular format. In addition to the conventional features such as DBH (m), Tree height (m), and Crown Projection Area (*m*^2^), we measured of the crown projection radius in 72 different directions, with each direction spaced 5 degrees apart in a 360-degree span (see Table 2 in Data Records section). Moreover, we measured the crown radius in 72 different directions at 2-meter intervals, starting from the tree base and extending up to 30 meters in height (Fig. 7). To accurately locate each tree within the project, we extracted the tree’s project location information and incorporated it into the dataset. Additionally, we transformed these project location coordinates to their corresponding global coordinates using the transformation matrix (Eq. 1) specific to each project. Consequently, the dataset includes two sets of location coordinates for each tree: the tree’s location within the project and its corresponding geographic coordinate system coordinates. In order to label the trees with their botanical names, we manually identified their species using Google Street View. Furthermore, we conducted thorough field visits to verify and ensure the accuracy of the botanical name labels in the dataset.

**Table 2:** The “TreeML_Dataset.csv” file contains the following column features for each tree.

features	explanation
date	signifies the date of the scanning process (see Data Records section)
projectID	a unique set of numbers assigned to each project (see Data Records section)
treeID	a unique ID for each tree in dataset (see Data Records section)
botanical.name	the botanical name (species) of the tree
location.x, location.y, location.z	x,y,z-coordinates of the starting point of the tree in project coordinate system
location.lat, location.long, location.alt	x,y,z-coordinates of the starting point of the tree in global coordinate system
DBH(m)	the diameter of the circle fitted to the height 1.36-1.33 m
treeHeight(m)	Height (m) of the tree
crownStartHeight(m)	Crown’s base height (m) from the ground
crownDiameterMax(m)	Maximum horizontal crown diameter (m)
crownProjectionArea(*m*^2^)	Area (*m*^2^) of the crown’s planar projection’s convex hull
totalVolume(L)	Total volume (L) of the tree
crownX_XXd_m_	crown radius in 72 directions, each 5 degree (m)
cr_XXmX_XXd_m_	crown radius in 72 directions, each 5 degree, and each 2 meter hight (m)

### TreeML-Structure Measurement

**Fig. 8:**
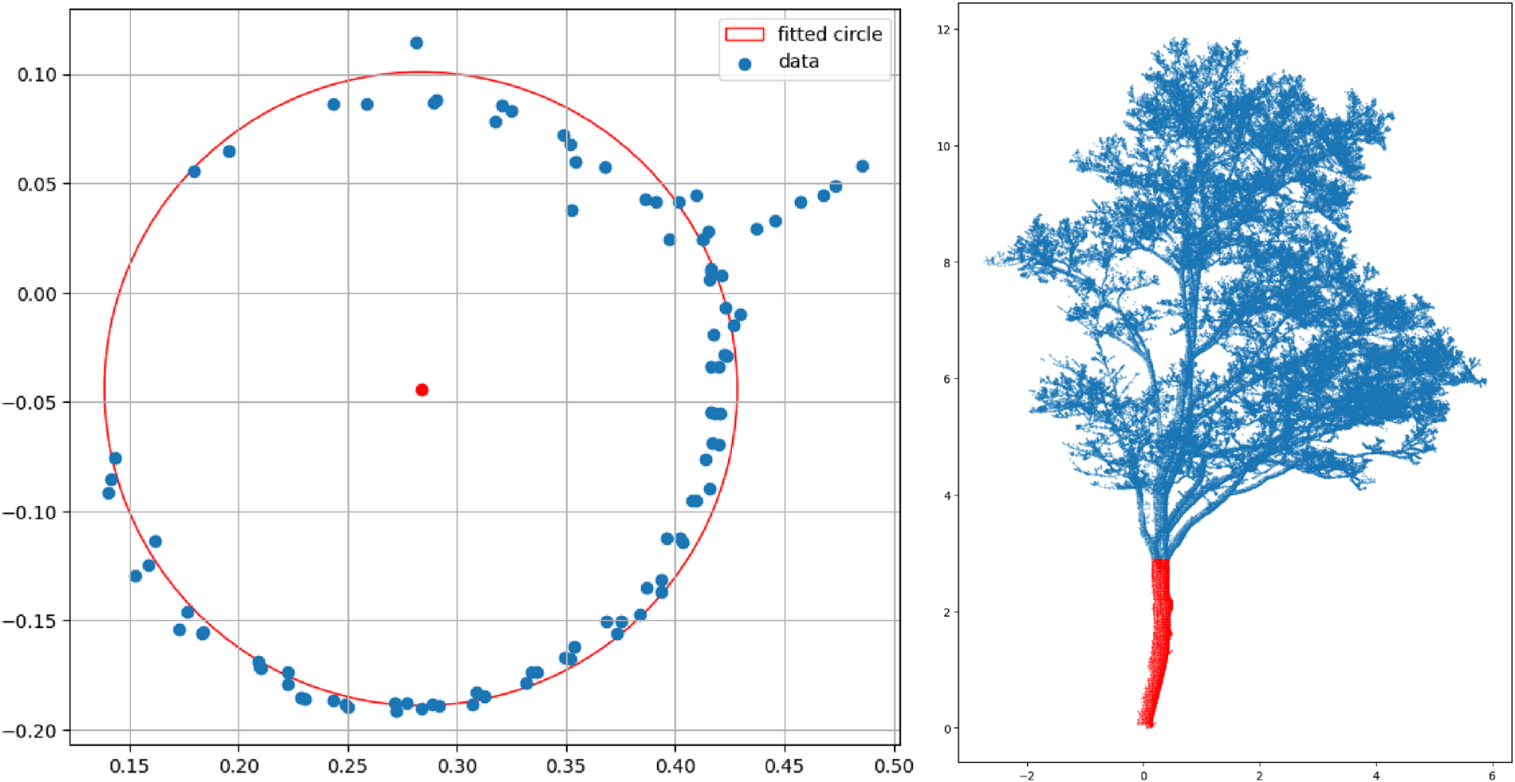
The circle fitting method in the TreeML-SM model was utilized to determine the Diameter at Breast Height (DBH) of trees (left). A segmentation of the trunk and crown points with the TreeML-SM method (right).

Despite utilizing TreeQSM for generating tree structure data, we encountered some errors in this step as identified during the quality check (see Data Technical Validation section). Consequently, we developed a custom Python script called TreeML-Structure Measurement (TreeML-SM) to extract the general tree structure based on the point clouds. This script generated all the features mentioned in Table 2 (see Data Records section), except for the crown radius in 72 different directions at 2-meter intervals from the trunk base to 30 meters. To enhance the accuracy of the point cloud data, we initially applied a noise and outlier detection method. Specifically, we employed a K-Nearest Neighbors Algorithm (KNN) from the sklearn library to measure the average distance between each point and its 20 closest neighboring points. Any point with an average distance greater than 30 cm was deemed an outlier and subsequently removed from the point cloud. For determining the tree location, DBH and crown start, we employed circle fitting methods. Firstly, we fitted a circle to the lowest 30 cm slice of the point cloud to identify the tree’s location. The center of the fitted circle represents the tree’s location within the dataset. Additionally, a circle was fitted to a 1.27 to 1.33-meter slice for defining the DBH (refer to Fig. 8, left). The resulting circle’s diameter provided the DBH. To determine the crown start height, we utilized the circle fitting method on 10 cm slices starting from the DBH height. We fitted a circle to each slice and identified the specific slice where the radius of the fitted circle was at least 20% larger than the radius of the previous circle. The height of this particular slice was recorded as the crown start height in the dataset (refer to Fig. 8, right).

**Fig. 9:**
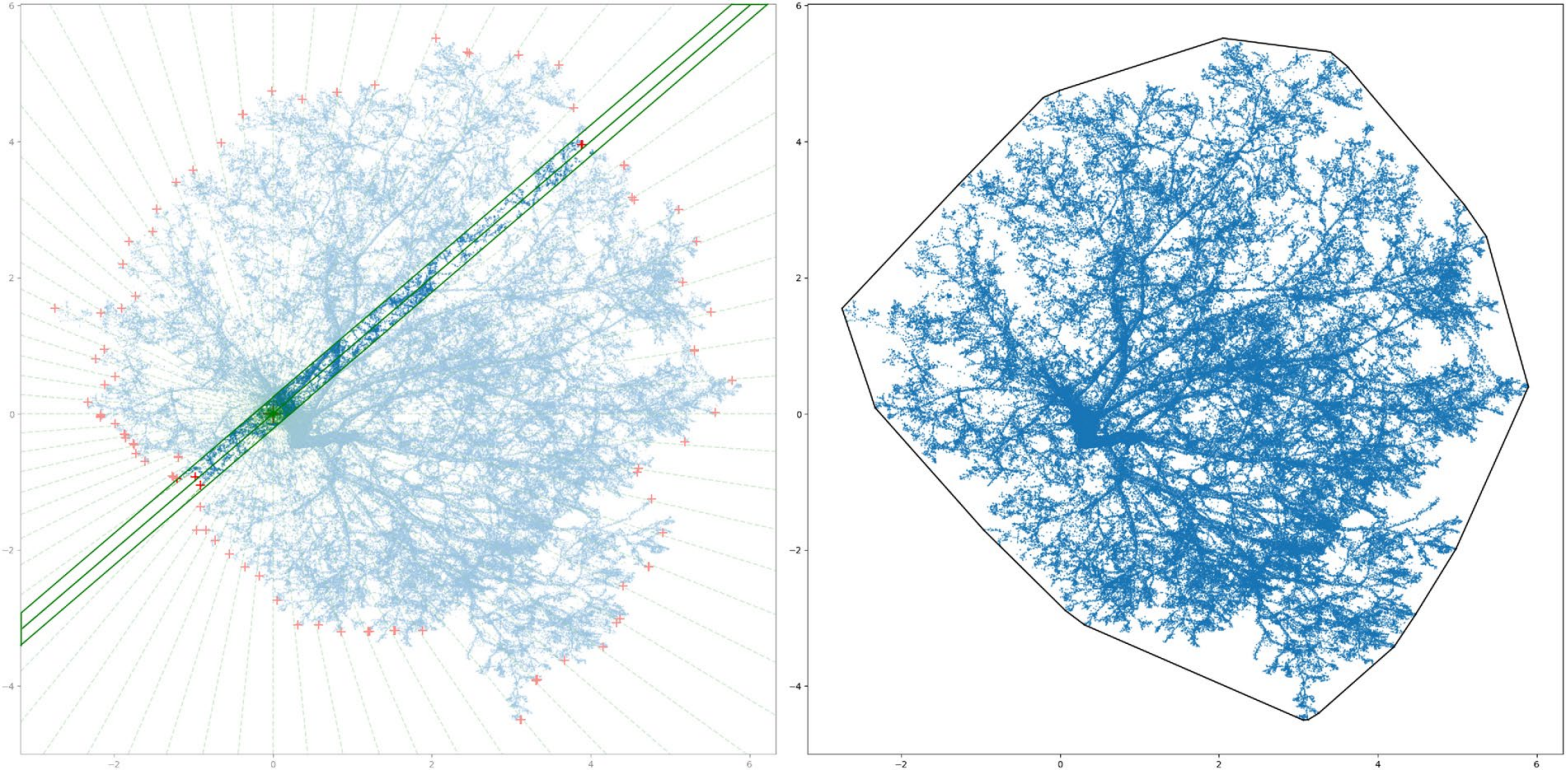
Defining the crown radius in each 5-degree increment (left). The crown projection area calculation with convex hull method (right).

Tree height was determined by calculating the vertical difference between the lowest point and the highest point within each tree’s point cloud. This measurement provided an estimate of the vertical extent of the tree. However, determining the crown diameter radius in each 5-degree interval was a more intricate task compared to determining tree height. To accomplish this, we sliced the tree’s point cloud vertically into 10 cm sections multiple times in each direction (see Fig. 9, left). For each slice, we determined the longest distance from the tree’s location to the points within the slice on the 2D plane. These longest distances were recorded as the crown radius values for each direction. Furthermore, we calculated the maximum diameters to determine the max crown diameter value within the dataset. To calculate the crown projection area, we employed the 2D convex hull method (http://www.qhull.org/) (see Fig. 9, right). By defining the convex hull of the crown on the 2D XY plane, we were able to calculate the area enclosed within the convex hull, which represents the crown projection area in the dataset. The TreeML-SM script is open source and can be accessed through the GitHub repository (https://github.com/hadi-yazdi/TreeML-Data).

### Graph structure modelling

**Fig. 10:**
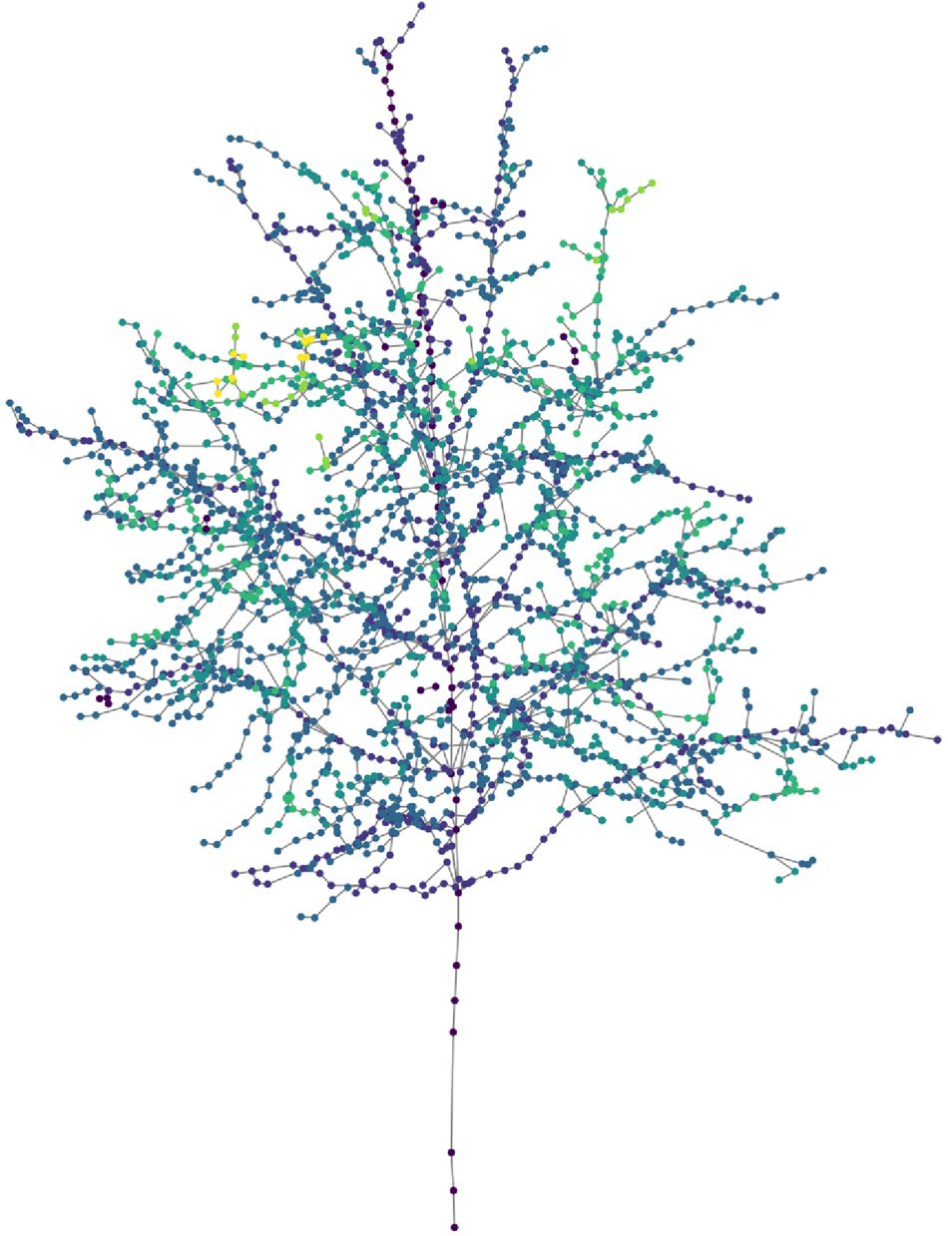
A graph structure model of the same tree in Fig. 6, where each node represents a cylinder and contains detailed structural information.

The final step of the TreeML-Data is the generation of a graph structure^19^ from the QSM data. Each cylinder of the trees contains structural information such as parentCylID, childCyID, start_x, start_y, and start_z (refer to Table 3 for a detailed overview). This dataset was used to generate the graph structures of the trees using the Networkx library (https://networkx.org/) in Python. To create the graph models, we divided the data into two tables. The first table consists of the cylinderID and parentCylID features, which represent the edges of the graph. The remaining features were saved in a separate table and assigned to the corresponding nodes. The generated graph models, comprising the list of nodes and edges, were saved in the dataset for further analysis. The Fig. 10 illustrates an example graph model for the same tree shown in Fig. 6, where each node represents a cylinder. The color of the nodes corresponds to the “branchOrder” feature. The saved graph files are in the “pickle” (https://docs.python.org/3/library/pickle.html) format, which is a binary system for serializing Python objects. The Python script for generating the graph structure from the QSM cylinder features and reading the “pickle” files is available on our GitHub repository (https://github.com/hadi-yazdi/TreeML-Data).

**Table 3:** The CSV files in the “optcsv” directory contain the following features for each cylinder.

features	explanation
date	signifies the date of the scanning process (see Data Records section)
projectID	a unique set of numbers assigned to each project (see Data Records section)
treeID	a unique ID for each tree in dataset (see Data Records section)
branchID	a unique ID for the branch of the cylinder (row number in the branch structure)
branchOrder	branch order of the branch the cylinder belongs
cylinderID	a unique ID for each cylinder in a tree (row number)
posInBranch	running number of the cylinder inside the branch it belongs
parentCylID	parent cylinder ID
childCyID	child cylinder ID
start_x, start_y, start_z	x,y,z-coordinates of the starting point of the cylinder
axis_x, axis_y, axis_z	x,y,z-components of the cylinder axis, unit vector
length	length of the cylinder
radius	radius of the cylinder
addedVirtual	if = 1, then the cylinder is added after normal cylinder fitting

## Data Records

The multidisciplinary and multilayer TreeML-Data^22^ is published in the Figshare repository. The data set contains six main files: “Dataset_pointcloud.zip,” “Dataset_Graph.zip,” “Dataset_QSM.zip,” “Dataset_transformation_matrix.zip,” “Projects_map.kml,” “TreeML_Dataset.csv,” and “TreeML_Dataset_QSM.csv.” These files encompass the main CSV dataset, labeled point clouds, individual tree point clouds, QSMs, and graph structure models. Within the dataset, there are 40 scanning projects, containing a total of 3,755 trees. The project names follow the format “project date”_“project ID,” where the “project date” signifies the date of the scanning process and the “project ID” is a unique set of numbers or names assigned to each project (e.g., “17_2_18” or “tum_campus”). For each tree, the point cloud or QSM files are labeled with a unique number appended to the project name, creating a filename format of “project date”_“project ID”_“Tree ID”. For instance, the file name of the tree featured in Fig. 6 is “2023-01-09_tum_campus_000003”.

### Tree Structure Data (.csv, .kml)

**Fig. 11:**
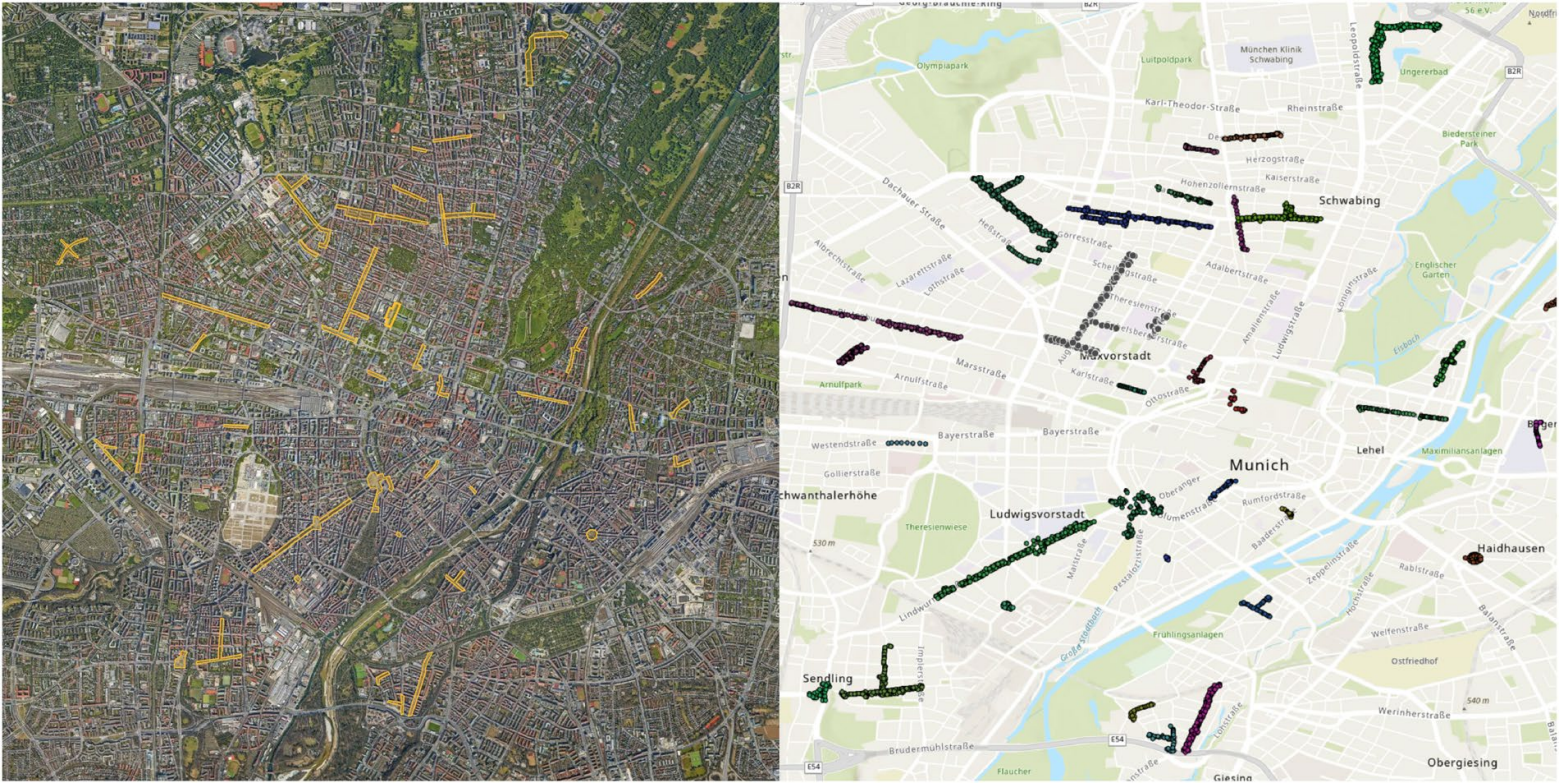
The geolocation visualization of the projects is viewed in Google Earth using the “projects_map.kml” file (left). The individual tree dataset’s geolocation is visualized using ArcGIS pro with “TreeML_Dataset.csv” file (right).

**Fig. 12:**
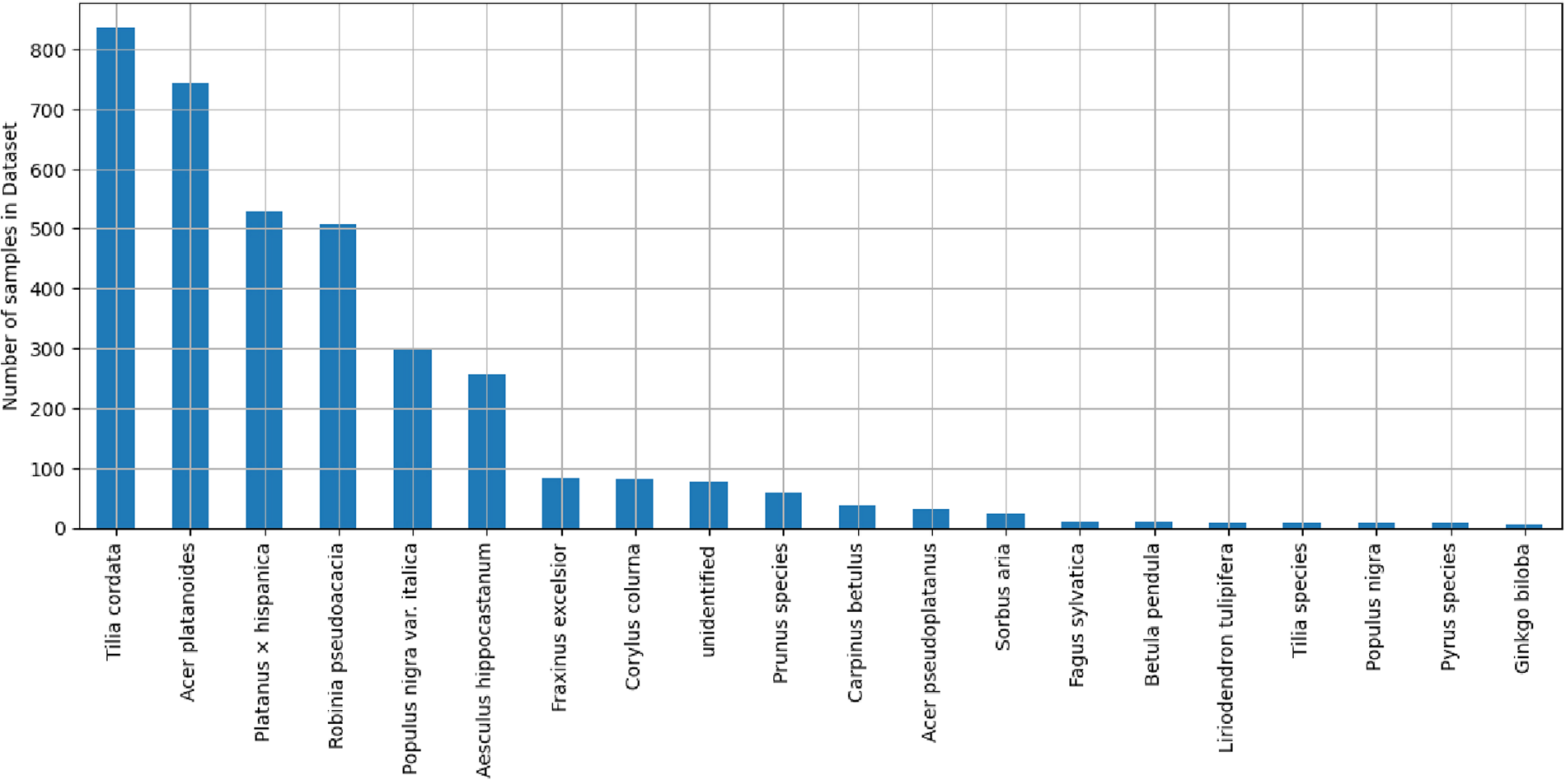
Number of samples of the 20 most frequent species in the dataset.

The projects map file is provided in .kml format, allowing users to view the locations of the 40 projects on Earth browsers such as Google Earth. This file serves as a guide for locating each project based on their respective project names (Fig. 11, left). The main CSV tree dataset, named TreeML_Dataset, contains the structural measurements (Table 2) of 3,755 trees, along with their corresponding botanical names (Fig. 12). Additionally, we have included a separate dataset CSV file named “TreeML_Dataset_QSM,” which presents the structural measurements of the entire tree dataset obtained through the TreeQSM model. We have made this dataset publicly available to document the tree structure measurements achieved using the TreeQSM model on the TreeML-Data, with the intention of facilitating future studies. To visualize the entire dataset, we utilized the global location coordinates available in the dataset (location.latitude, location.longitude, location.altitude) and displayed it in a geographic information system software, such as ArcGIS Pro (https://www.esri.com), as shown in Fig. 11 (right).

**Fig. 13:**
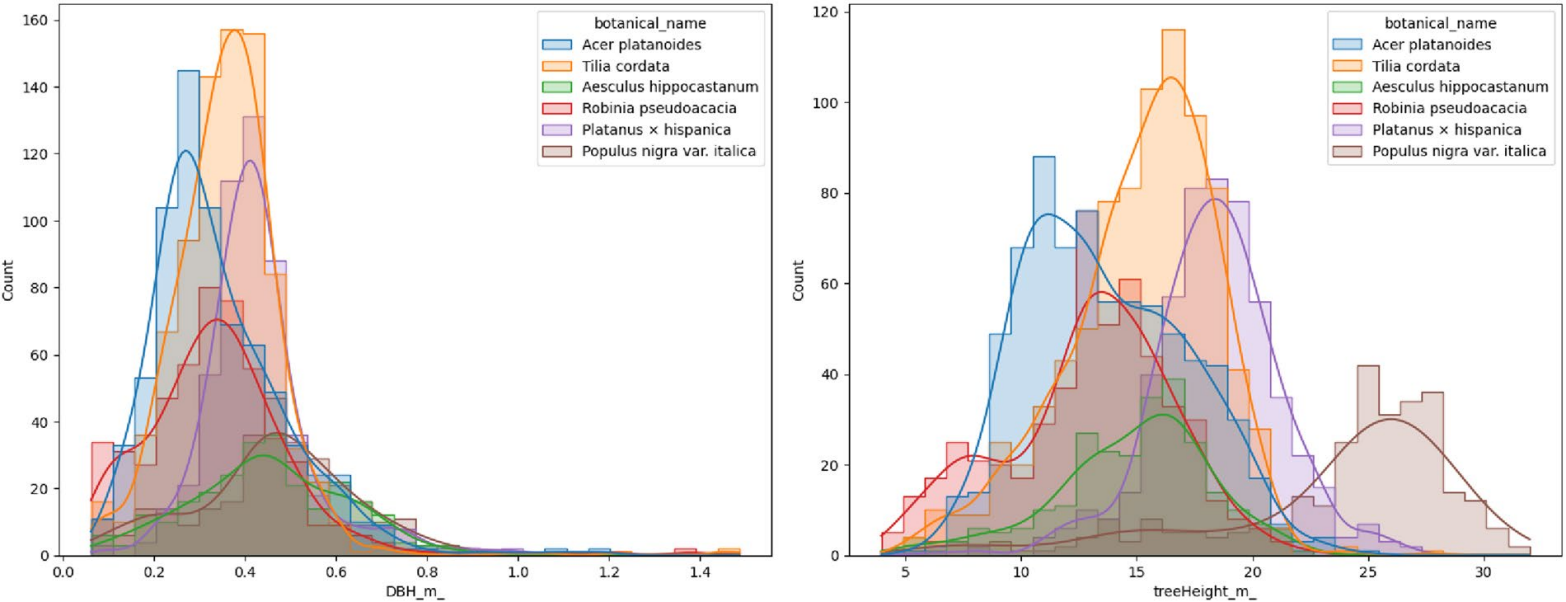
Tree count histogram by DBH (left) and tree height (right) for 6 most frequent species in the dataset.

The dataset contains multiple tree species, with Fig. 12 showcasing the 20 most common species that can be found in temperate Central European cities. There are also 82 trees in the dataset labeled as “unidentified”. These trees could not be easily identified during the labeling process or there was uncertainty in their classification. To maintain the dataset’s accuracy, they were categorized as unidentified. For visualization purposes, we focused on the six most frequent species. Figure 13 displays the histogram of their DBH on the left and Tree height on the right. These histograms provide insights into the distribution of DBH and Tree Height for the selected species.

### Labelled point clouds (.txt, .dat)

**Fig. 14:**
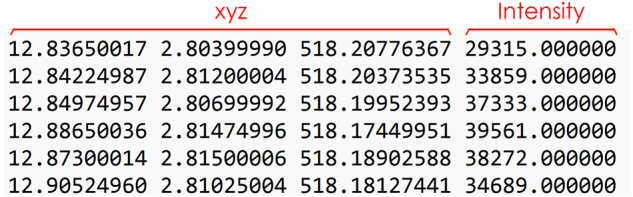
The point cloud files in the dataset follow a specific data structure. Each line in the file represents a single point in the point cloud, and it consists of four numbers. The first three numbers represent the location coordinates of the point. The fourth number in each line represents the intensity value of the point.

In the “Dataset_pointcloud.zip,” you will find two files related to the point clouds in the dataset: “Dataset_building_other.zip” and “Dataset_tree.zip.” The “Dataset_building_other.zip” file contains separate text files for each project, specifically for the “Buildings” and “Other” point clouds. On the other hand, the “Dataset_tree.zip” file includes all the point cloud files for the trees in each project. These files are in TXT format and consist of four main numbers representing each point in the point clouds. The first three numbers represent the location coordinates of the point. These coordinates typically correspond to the X, Y, and Z coordinates in a 3D space, indicating the position of the point within the project. The fourth number in each line represents the intensity value of the point. The intensity value provides additional information about the characteristics or properties of the point, which can be useful for certain analyses or visualizations (Fig. 14). The point cloud dataset comprises 3,755 individual tree point clouds, 40 building point clouds, and 40 other point clouds.

### QSM and graph data of individual trees (.mat, .csv, .pickle)

The “Dataset_QSM.zip” file includes three directories: “opt” “optcsv” and “trans” which correspond to each project in the dataset. The “opt” directory contains the main Quantitative Structure Model (QSM) files in “.mat” format. These files store the structural information of the tree cylinders, including their geometry and other relevant attributes. In the “optcsv” directory, you can find the extracted features from the QSM files in a more accessible format, specifically as “.csv” files. These files contain the selected features of the cylinders, as described in Table 3, making it easier to work with and analyze the QSM data. Lastly, the “trans” directory holds the transformation information files. These files provide the necessary details for converting the location coordinates of the cylinders to the project’s coordinate system. The “Dataset_Graph.zip” file contains the graph models of the trees in the dataset. These graph models are saved in the “pickle” format, which is a binary format used for serializing Python objects. The graph models capture the structural information and relationships of the cylinders in each tree, representing the hierarchical organization of the branches.

## Technical Validation

**Fig. 15:**
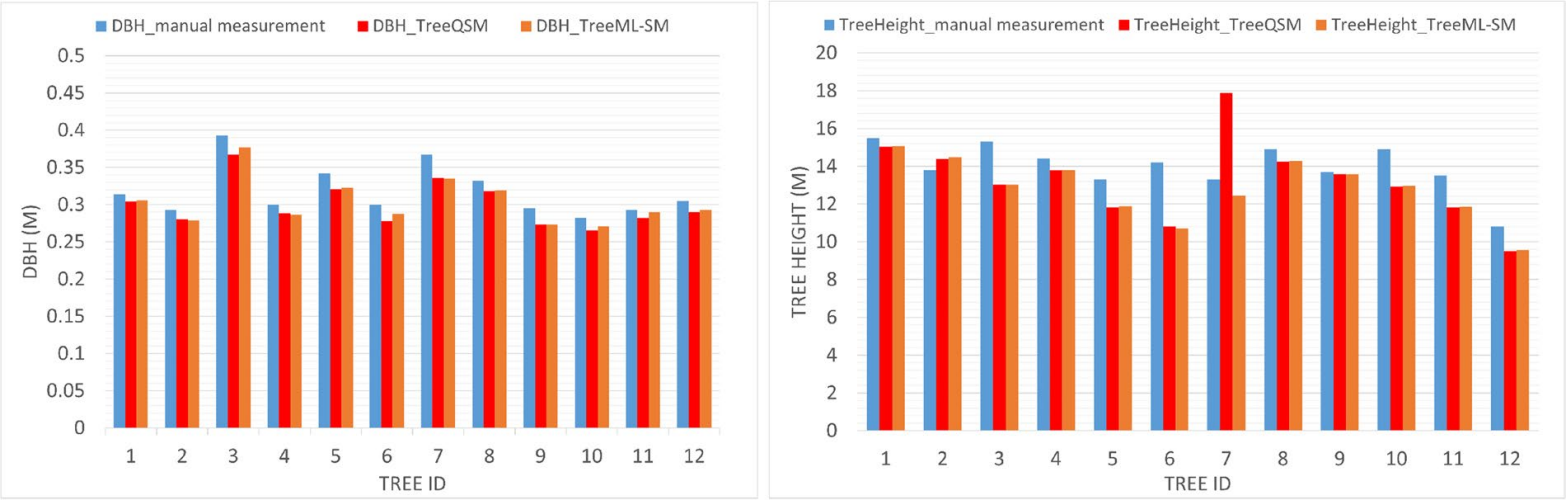
The figure shows the comparison of hand measurement (blue), TreeQSM (red), and TreeML-SM (orange) on 12 sample trees in the dataset. On the left, we have the DBH (m), and on the right, we have the tree height (m).

**Fig. 16:**
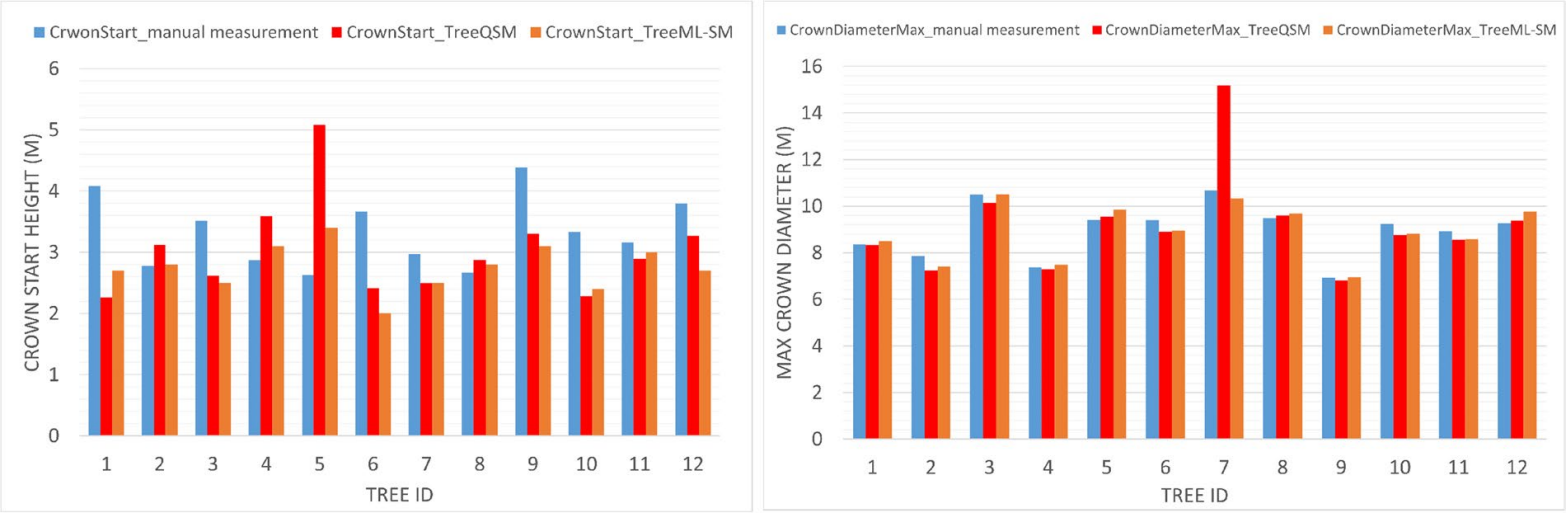
The figure shows the comparison of hand (blue), TreeQSM (red), and TreeML-SM (orange) on 12 sample trees in the dataset. On the left, we have the CrownStart(m), and on the right, we have the CrownDiameterMax(m).

To assess the accuracy and feasibility of the results, we conducted manual measurements on 12 trees and compared them with the dimensions derived from the TreeQSM and TreeML-SM. The manual tree structure measurement is known to have a relative high deviation, particularly in measuring tree height and crown size. However, manual DBH measurements is considered a solid reference to the digital methods like TreeQSM and TreeML. We compared the DBH, tree height, crown start, and maximum crown diameter measurements between the manual, TreeQSM, and TreeML-SM to evaluate their quality. For DBH measurements, we observed an average of 1.8 cm deviation between TreeQSM and manual measurements for these 12 trees. This deviation was reduced to 1.5 cm with the TreeML-SM (Fig. 15, left). This 1.5 cm difference can be attributed to two factors because the manually measured DBH is systematically higher than the QSM and TreeML-SM. Firstly, the manual measurement may not always be exactly at the height of 1.3 meters, as it was roughly estimated. Secondly, the point cloud segmentation step may lead to some loss of points at the start position of the trunk, causing the tree start points in the point clouds to be higher than the actual street level in the manual measurement, resulting in DBH measurements in point cloud being taken at a height higher than 1.3 m. The situation is different in measuring tree height, crown start, and max crown diameter. For them, the manual measurements are not robust enough to serve as a reference for assessing the accuracy of the digital measurements. In particular, the Nikon Forestry Pro height measurement (https://www.nikon.de) tool was used for tree height and crown start measurements. Figures 15, 16 illustrate the manual measurements in light blue, the TreeQSM in red, and the TreeML-SM in orange. We can observe two notable outliers in TreeQSM data: tree number 7 has an overestimated tree height and max crown diameter, and tree number 5 has an error at the crown starting hight. Upon inspecting the point clouds, we identified these two significant errors in the TreeQSM for these two samples. These errors motivated the development of the TreeML-SM to enhance the accuracy of the final dataset. Notably, in Fig. 16 (right), the manual, QSM, and TreeML-SM measurements, except for tree number 7 in QSM, closely align with each other despite the low accuracy of the manual measurement method.

**Fig. 17:**
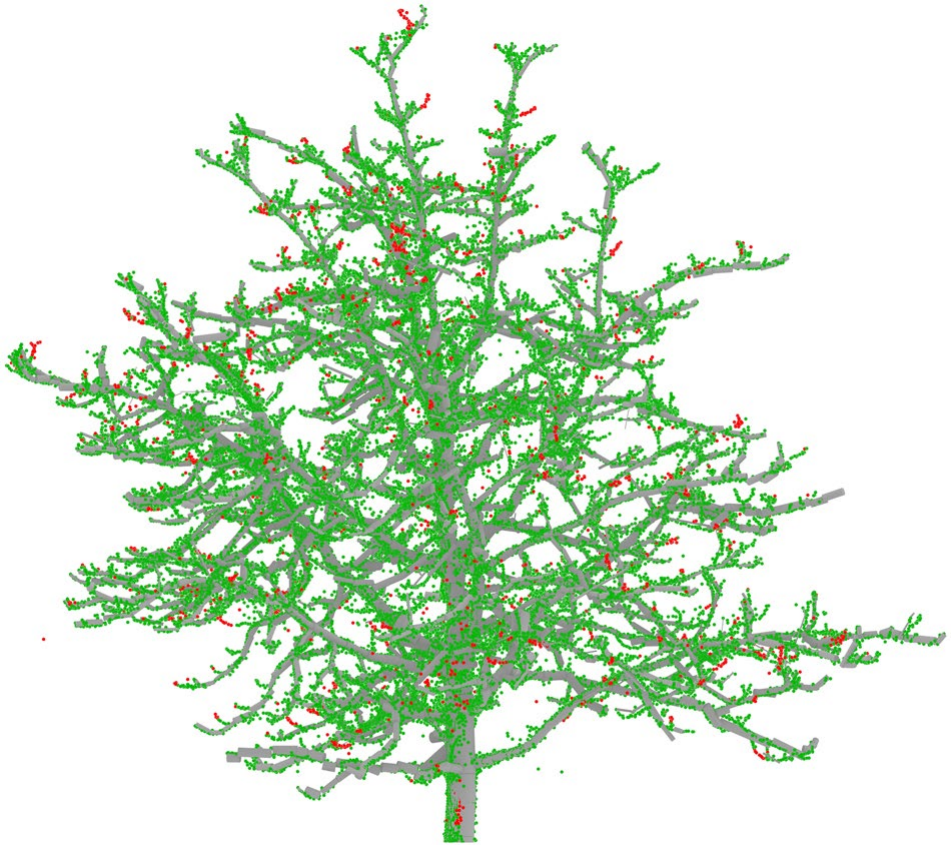
The quality check of the cylinder fitting model was performed using the point cloud of a sample tree. The cylinder fitted points are represented by the green color, while the red points indicate small twigs that were not fitted with any cylinder.

Next, we conducted a quality check of the TreeQSM cylinder fitting model. In this step, we calculated the distance between each point and the closest cylinder. We then identified points with a significant distance from the cylinder as “not fitted” points. To assess the feasibility, we visualized these points and compared them with the cylinder fitting model. Figure 17 illustrates this visual check using a sample tree from the dataset. The points fitted with a cylinder are shown in green, while the red points indicate those that were not fitted with any cylinder in the QSM model. These red points mainly consist of small twigs at the end of the branches, which are too small to be considered as branches suitable for cylinder fitting. This quality check demonstrates that most branches are successfully fitted by cylinders, with only a small number of twigs being missed in the final QSM models. However, it should be noted that the accuracy of the fitted cylinder’s diameter cannot be determined using this method.

## Usage Notes

TreeML-Data is a multidisciplinary and multilayer dataset that can be utilized by researchers from various disciplines for validation and development purposes. It encompasses high-precision labelled point clouds and isolated urban trees, which are valuable for remote sensing, computer science, and machine learning researchers. The tree measurement, QSM data, and associated Python scripts provide valuable resources for urban forestry, green architecture, and tree specialists. Additionally, the graph structure data introduces a novel approach to tree information, opening new avenues for research. For example, these Tools can be tested with our dataset as a benchmark: (1) urban tree Segmentation (we have tested PointNet++); (2) tree instance segmentation for automated isolating of the trees in the point clouds; (3) QSM reconstruction (treeQSM is used in these study); (4) tree structure measurement (we developed own TreeML-Structure Measurement model); (5) Urban tree allometric equations (the tabular urban data dataset can be used for testing allometric equations). As far as our knowledge extends, TreeML-Data is the largest open-source tree graph structure dataset. We have made the process of gathering the dataset easily accessible by publishing the corresponding codes on GitHub repository.

## Data Availability

The TreeML-SM script “TreeML-SM.py”, transformation script “point_transformation.py” for location transformation from project coordinate system to global coordinate system, and pre-trained point cloud segmentation model are published in the GitHub repository (https://github.com/hadi-yazdi/TreeML-Data). Please refer to the Readme file in the Github repository for further information.
